# Curriculum Innovation: Combining Didactic and Clinic-Based Methods to Improve Palliative Care Education for Neurology Residents and Advance Practice Providers

**DOI:** 10.1212/NE9.0000000000200249

**Published:** 2025-09-23

**Authors:** Andrew P. Huang, Cindy Gibson, Sandhya Seshadri, Benzi M. Kluger

**Affiliations:** From the Department of Neurology, University of Rochester Medical Center, NY.

## Abstract

**Background and Objectives:**

The American Academy of Neurology recognizes a need for neurology clinicians to provide some aspects of palliative care. Palliative care is person-centered and family-centered care that focuses on quality of life for persons living with life-limiting illness. While palliative education programs exist, there is a knowledge gap regarding how to integrate this education into real-world neurology practice. The aim of this study was to improve the perceived knowledge of, comfort with discussing, and frequency of discussing palliative care topics in outpatient neurology practice.

**Methods:**

Recorded didactic lectures, note templates, and live meetings to discuss real-world challenges were implemented at an academic neurology department and offered to residents and advanced practice providers (APPs). A sample of residents and APPs completed surveys before and 6 months after using this curriculum. Surveys asked questions with Likert responses about their knowledge of, comfort with, and frequency of discussing 7 palliative care topics. A sample of learners and supervising faculty participated in semistructured interviews where authors explored their perceptions about palliative care and this curriculum. Interviews were audiotaped, transcribed verbatim, deidentified, coded, and analyzed.

**Results:**

A total of 19 clinicians completed pre-education surveys and 12 completed posteducation surveys. Surveys showed statistically significant improvements overall, including in practical support for patients/care partners, discussing complex symptom management, advance care planning, higher levels of care, and discussing disease course. Overall, 12 interviews were completed, with 4 themes identified: (1) *Practical curriculum*: the curriculum connected theory to practice; (2) *Palliative mindset:* the curriculum gave recognition of the suffering of neurologic illness; (3) *First, do no harm:* clinicians worried about how palliative discussions could harm patients and families; (4) *Systemic barriers*: neurology clinicians faced systemic barriers in addressing palliative care.

**Discussion:**

Neurology clinicians want to practice palliative care but need ongoing education, sufficient time, clinical availability, and supervisory support to do so. Lectures, note templates, and live meetings to practice palliative care can be implemented feasibly in neurology practice and show preliminary signs of efficacy. Further work is needed to develop and test methods that aim to influence real-world practice.

## Introduction

Worldwide, neurologic illness is a leading cause of morbidity and mortality.^1^ Since 1996, the American Academy of Neurology^2,3^ has recognized a need for neurologists to provide some aspects of palliative care. Palliative care is person-centered and family-centered care that focuses on quality of life for persons facing life-limiting illness.^4^ Educational programs exist that teach serious illness communication^5,6^ or palliative care in neurology.^7-12^ However, existing neurology educational programs do not yet routinely teach palliative care with practice-based approaches. Furthermore, little is known about how to integrate this education in real-world neurology practice.

### Objectives

The objective of this study was to improve the perceived knowledge of, comfort with discussing, and frequency of discussing palliative care topics in outpatient neurology practice.

## Methods

We conducted a sequential, mixed-methods study using questionnaires, followed by semistructured interviews to assess the feasibility, preliminary efficacy, and potential barriers of a curriculum that used 3 educational strategies: recorded lectures, live meetings, and note templates.

### Study Setting and Participants

This study was conducted at the Department of Neurology at the University of Rochester Medical Center. A total of 37 neurology residents and advanced practice providers (APPs) were eligible to participate in the study over 2 years. In 2021, the curriculum was offered only to PGY3 and PGY4 residents and APPs who expressed interest in the education (N = 15). In 2022, the curriculum was offered broadly to neurology residents (PGY2–4) and APPs in the department (N = 22). Because we were interested in single-exposure effects, we excluded 4 participants who completed surveys in 2021.

### Curriculum

Participants underwent 3 simultaneous strategies to teach palliative care:

1. Recorded didactic lectures: Participants were asked to watch 3 recorded lectures that were developed and presented by neurology and palliative care faculty. Two topics were required (“Introduction to Neuropalliative Care” and “Advance Care Planning”), and participants had the option to select other topics listed in eAppendix 1 (e.g., serious illness communication, difficult emotions, integrating palliative care into practice, neuropalliative hospice, neuroprognostication, non–pain symptom management, care partner assessment). Freely available didactic content is offered online by the International Neuropalliative Care Society, including PowerPoint modules^13^ and Speaker Series panel interviews. Recorded lectures can be requested from the first author (A.P.H.).
2. Live meetings: Two live meetings took place each year. Participants were asked to attend at least 1 live Zoom meeting to discuss real-world implementation (e.g., use of note templates). The purpose of these meetings was to create a nonjudgmental space for learners to discuss challenging clinical cases, difficult conversations, approaches to palliative care discussions, and strategies for integrating palliative approaches into their care. These meetings were facilitated by authors A.P. Huang (palliative care fellow, neurologist) and B.M. Kluger (neurology faculty, palliative care researcher) without a facilitator guide or agenda, centering on learners' questions and experiences rather than specific topics such as the didactics.
3. Note templates: In the electronic medical record, participants were asked to use palliative care–focused note templates (eAppendix 2) in their clinical settings (e.g., general neurology resident clinic or subspecialty clinics). These note templates were adopted from senior author B.M. Kluger's template from a previous clinical trial.^14^

## Method of Assessment

### Surveys

Participants were asked to complete surveys before and 6 months after using the curriculum. These voluntary surveys feature questions with Likert scales (eAppendix 3, score of 1–5) about perceived knowledge of, comfort with discussing, and frequency of discussing 7 palliative topics: spiritual distress, psychiatric concerns, complex symptom management, practical support for patients and care partners, higher levels of care, discussing disease course/complications, and advance care planning. Surveys were developed and piloted with members of an advisory council of faculty, residents, and APPs involved in research and education in palliative care. This council reviewed literature of surveys in teaching palliative care in oncology^15^ and optimized wording to ensure coverage of key topics. Presurveys and postsurveys were not paired, resulting in some participants only completing presurveys or postsurveys.

Wilcoxon rank-sum (Mann-Whitney) tests were used to compare presurvey and postsurvey scores in knowledge of, comfort with discussing, or frequency of discussing each palliative topic. Category sums (ranging from 7 to 35) and survey sums (21–105) were calculated. Wilcoxon rank-sum tests were used because samples were unpaired and normal distribution was not assumed. We did not account for multiple comparisons.

### Semistructured Interviews

We then completed semistructured interviews using a qualitative methodology^16^ with residents and APPs who completed the curriculum and with faculty who precepted residents in the continuity clinic. This convenience sample was based on individuals who responded to emails that requested their opinions and perceptions. Interview guides were developed and pilot tested with an advisory team of faculty, residents, and APPs and used for all participants. Questions asked why learners went into neurology, perceptions of the curriculum, how they encountered palliative care issues in their practice, and barriers to palliative care (eAppendix 4). Participants knew that the interviewers were palliative care champions and advocates. Most of the interviews were completed by A.P. Huang (palliative care fellow at the time). Interviews were audio-recorded, transcribed verbatim, and deidentified to maintain confidentiality. Transcripts were not returned to participants for feedback. The Consolidated Criteria for Reporting Qualitative Research (COREQ) checklist for qualitative research was used.

Qualitative data were analyzed inductively^17^ with open coding. A codebook was developed iteratively as a group (authors A.H., S.S., and C.G.) as interviews were reviewed, resulting in 9 codes. The research team comprised author A.P. Huang, male palliative care fellow; S. Seshadri, female qualitative research faculty; C. Gibson, female neurology APP; and B.M. Kluger, male neurology research faculty. While A.P. Huang was new to qualitative research, C. Gibson, S. Seshadri, and B.M. Kluger had >10 years of experience in qualitative research. While A.P. Huang and C. Gibson knew many participants before the interview, S. Seshadri and B.M. Kluger knew very few of the participants. This team analyzed the coded interviews and identified themes using Atlas.ti. After 10 interviews, we did not identify new themes related to the study questions. We conducted 2 additional interviews to ensure that we had adequate data for our study aims.

### Standard Protocol Approvals, Registrations, and Participant Consents

This study was reviewed and deemed by the Institutional Review Board at the University of Rochester Medical Center to be exempt from requiring consent (URMC Research Subjects Review Board STUDY00007044).

### Data Availability

Data can be made available on request from the corresponding author.

## Results

### Survey Participants

A total of 37 clinicians participated in the curriculum over 2021 and 2022. Of those, 19 and 12 clinicians completed pre-education and posteducation surveys, respectively. In 2021, 10 and 8 participants completed before education and after education surveys, respectively. In 2022, 9 and 4 participants completed before education and after education surveys, respectively. Residents were 37% female and ranged from PGY2 to PGY4, with a median age of 29 ± 4 years. All APPs were female and had more than 10 years of clinical experience, with a median age of 47 ± 13 years. Participants reported watching at least 3 lectures. However, the number of total lectures watched, live meeting attendance, and note template use were unmeasured.

### Self-Assessment Survey Results

Overall, the average survey score (range 21–105) increased by 26% (15.3 points), from 59.9 before education to 75.2 after education (*p* = 0.02, 95% CI [1–28]) (Table 1). Category sum scores (range 7–35) also increased in *perceived knowledge* (19.7–25.2, *p* = 0.02, CI [1–10]), *comfort discussing* (21.5–27.2, *p* = 0.01, CI [1–10]), and *frequency discussing* (18.6–22.8, *p* = 0.08, CI [−1 to 9]). Efficacy of education was highest in *practical support for patients and care partners*, *advance care planning*, *discussing disease complications*, and *complex symptom management.* After education survey scores were not statistically increased in *existential/spiritual distress* and *psychiatric/mood concerns*. Table 1 provides specific topics.

**Table 1:** Survey Results of Perceived Knowledge of, Comfort With Discussing, and Frequency of Discussing Palliative Care

	Reported perceived knowledge			I feel comfortable discussing			How frequently you discuss		
	Before, N = 19	After, N = 12	Wilcoxon rank-sums^a^	Before, N = 19	After, N = 12	Wilcoxon rank-sums	Before, N = 19	After, N = 12	Wilcoxon rank-sums
Survey score^b^ (21–105)	59.9	75.2	0.02 (1, 28)						
Category sum score (7–35)	19.7	25.2	0.02 (1, 10)	21.5	27.2	0.01 (1, 10)	18.6	22.8	0.08 (−1, 9)
Existential or spiritual distress	2.53	3.00	0.16 (0, 1)	2.74	3.5	0.11 (0, 2)	1.79	1.83	0.95 (−1, 1)
Psychiatric/mood concerns	3.11	3.50	0.10 (0, 1)	3.47	3.83	0.64 (0, 1)	3.32	3.58	0.46 (−1, 1)
Complex symptom management	2.79	3.50	0.04 (0, 2)	3.16	3.75	0.08 (0, 1)	2.68	3.50	0.04 (0, 1)
Practical support for patients/care partners	2.53	3.75	0.01 (0, 2)	2.89	4.00	0.02 (0, 2)	2.74	3.75	0.04 (0, 2)
Higher levels of care	2.68	3.67	0.03 (0, 2)	2.79	3.92	0.006 (0, 2)	2.42	3.00	0.22 (0, 2)
Discussing disease course/complications	3.05	3.83	0.02 (0, 1)	3.21	4.08	0.02 (0, 2)	3.37	4.00	0.07 (0, 1)
Advance care planning	3.05	3.92	0.04 (0, 2)	3.26	4.08	0.01 0, 1	2.32	3.17	0.08 (0, 2)

a Brackets include 95% CIs for Wilcoxon rank-sums.

b Note that scores throughout are means: 1 = little or no…, 3 = moderate…, 5 = advanced perceived knowledge, comfort discussing, or frequency discussing.

### Interview Participants

Our research team (1 faculty, 1 resident, 1 advance practice practitioner, 1 fellow) contacted 10 residents, 8 advance practice practitioners, and 8 faculty. We completed 1-on-1 qualitative interviews with 4 faculty, 4 APPs, and 4 residents (2 in PGY3, 2 in PGY4) through Zoom or in person at nonclinical locations, lasting 25–60 minutes. Field notes were not recorded. We did not ask why individuals turned down participation. All faculty precepted in resident clinic. APPs had more than 10 years of clinical experience.

### Interview Results

Our team identified 2 themes related to facilitators, *Practical curriculum* and *Palliative mindset*, and 2 themes related to barriers toward practicing palliative care, *First, do no harm* and *Systemic barriers* (Table 2).

**Table 2:** Two Themes Regarding Facilitators and 2 Themes Regarding Barriers

Theme	Subthemes
1. Practical curriculum	1. Didactic lectures gave bite-sized chunks of information
	2. Live meetings allowed discussion of real-world issues and practice skills
	3. Note templates gave organization to palliative encounters
2. Palliative mindset	1. The curriculum taught the palliative approach to care
	2. The curriculum motivated clinicians to address palliative care in their practice
3. First, do no harm	1. Clinicians fear making a “bad scene”
	2. Palliative discussions can feel like a “can of worms”
4. Systemic barriers	1. Palliative care conversations take time
	2. Clinicians lack follow-up availability
	3. Extracurricular palliative education was suboptimal
	4. Some attendings did not prioritize palliative care

### Theme 1: Practical Curriculum

Participants felt that the curriculum connected theory to practice (Subtheme 1). Didactic lectures gave bite-sized chunks of information:

I loved the medium that… we could access it… They were really good… bite sized chunks… [It let me]… be reminded and refreshed on some of these ideas and topics. Uh, ideas of how to implement it… and really incorporate it more into my practice…” (Resident)

Many participants appreciated that the lectures were short, available on demand, and practical. Lectures communicated big picture concepts about palliative care such as the goals of medicine.

Participants felt that *live meetings allowed individuals to discuss real-world issues and practice skills* (Subtheme 2):

I definitely think [the meetings] provided tangible words, language considerations… that make me more comfortable to do that… and those tools are very helpful. (Resident)

I think [the live meetings] were most helpful “cause I got to actually think about … the palliative care mindset… Like, that really helped me a lot, and just thinking about and applying… strategies for patients. (Resident)

Some participants felt that the meetings offered language and strategies for conducting palliative discussions. Beyond didactic lectures, these meetings gave a learning space to discuss how to bring these topics up for patients and families.

Interviewees reported that *note templates gave organization to palliative encounters* (Subtheme 3):

Documentation… can get in my way of having a meaningful conversation… a necessary evil… so having structured templates like the palliative template, and then other… review of systems or… different disease areas to keep some questions relevant but more closed ended… to then… more open-ended questions… I think is helpful. So I really appreciate the template. (Resident)

Note templates provided “training wheels,” giving example questions and topics for when palliative topics arose. The note template provided organization that complemented the theory in lectures and language practice in live meetings.

### Theme 2: Palliative Mindset

Participants felt that *the curriculum taught the palliative approach to care* (Subtheme 1):

One patient… had a fairly rapid progression… [of] an atypical parkinsonian syndrome. At one point… he just looked at me and said “what does the future look like for a person like me?” And I thought, “Oh, ****. I don’t wanna tell you… I said to myself, how can we connect to your strength and to who you are as a person? Because the disease is secondary. (APP)

Most participants reported examples of patients who had symptoms and prognostic signs where the palliative approach was important. Faculty noticed that residents frequently had a hard time seeing the big picture for patients:

When [residents] think of palliative care, they think hospice, like end of life, goals of care… MOLST forms, all that kind of stuff. But I really feel like palliative care is really more like patient values… It doesn’t mean they’re imminently dying… it’s kind of hard to be like, “when you put those 20 body systems together and get a full human being… where are we?”… It’s hard to pull back and [say] we need a different approach. (Faculty)

Supervising neurologists emphasized the whole person and family approach of palliative care and how this approach could contrast the neurologic focus of residency training.

Furthermore, *the curriculum motivated clinicians to address palliative care in their practice* (Subtheme 2):

At the time, it might have been good to bring up talking about goals of care… I’m seeing that patient in a couple months. So I might bring that up then. (Resident)

I think the biggest thing is overall I’m… very thrilled that this project is being implemented, and I think in the future of neurology, neuropalliative care is something that should be… an even stronger piece in neurology and there should be hopefully some formalized training in it for all future neurologists. (Resident)

Through engagement with the curriculum, many clinicians imagined how they could address palliative care in their practice, even transforming 1 participant into a palliative care advocate.

### Theme 3: First, Do No Harm

Clinicians worried about how unwelcome palliative discussions could harm patients and families. *Clinicians feared making a* “*bad scene*” (Subtheme 1):

It’s almost like I created… a bad scene because people—I mean they never really had discussed any of the topics that I brought up. And they-they were hurtful… I just thought it was better for the patient to be able to, at least have these issues introduced, so then they could think about them.” (APP)

I had a new patient… I was really worried that patient had Huntington’s… I asked… what do you think this is? What are your biggest concerns?.… Ultimately the patient was… upset about the visit and… didn’t wanna see or follow with me every again…I’ve thought a lot about that visit…[was] I… confusing, not well-articulated, difficult to follow, tangential, or circumferential?… Should I have withheld information and not mentioned anything?… It was one of the most interesting and thought-provoking clinic patients that I had had. (Resident)

Many clinicians recalled experiences where talking about serious illness did not go well. While suboptimal delivery by early learners could play a role, patients may experience distress even amid tactful delivery.

Participants discussed how *palliative discussions can feel like opening a “can of worms”* (Subtheme 2):

I feel like I would be willing to explore [palliative needs] if I had time to do it… if I open this can of worms… I feel weird. I know it’s, like, not quite abandoning but… If we can’t… do a good job with it… it’s better… not to do it… [if we are not] following up with them… I feel weird… opening that too much. (Resident)

When neurology clinicians entered palliative conversations, they may have been unsuccessful because they lacked the skills or training to address the issue. It was dissatisfying to enter these discussions unequipped to address the issue.

### Theme 4: Systemic Barriers

In addition, neurology clinicians stated that they face systemic barriers in addressing palliative care. For example, they stated that *palliative care conversations take time* (Subtheme 1):

It’s just not having the time to… even bring up that discussion… You don’t wanna… be like, “Oh, hey, you should really think about what you would like in case you, you know, stop breathing. Um, I’m gonna go now”… you can’t just, like, drop it and leave. (Faculty)

If you're talkin' about somethin' sensitive like… have you ever heard of a MOLST form and what that means… it deserves more time… You can't just… say, oh, what's your code status, in five minutes and then keep goin' with the visit. …These topics are so sensitive, they deserve more attention, and they deserve easing into it. (Resident)

Residents, APPs, and supervising faculty alike reported inadequate time in their clinical settings to address palliative topics. Faculty and APPs who had more clinical experience especially recognized how time was a barrier. Preference-sensitive discussions rely on eliciting values from patients, and rushing them risks poor execution.

When recognizing palliative needs, *clinicians lack follow-up availability* (Subtheme 2):

Staffing decisions are made based on a best-case scenario… we can barely squeak by with the staffing that we’ve got if everything goes perfectly, but the reality is that it doesn’t… Many of the providers in my division have young children. We are always out… I’m fully booked for a couple months, you know? (APP)

We get four clinics a month… at least once per month, there’s a patient who absolutely deserves a visit just strictly [for palliative care]. (Resident)

Residents and APPs both felt overbooked and understaffed in their clinics to see patients in a timely fashion to allocate the time that palliative care topics require. No matter the quality of teaching palliative care in neurology, lack of follow-up availability can be a barrier.

Furthermore, *extracurricular palliative education was suboptimal* (Subtheme 3):

[How much of the curriculum have you seen?] I’m gonna be honest, like, what we’ve done in lectures and like practice… has probably been the most of… it… I found it personally to be tough to find the time to do that. (Resident)

I thought it was an important enough topic to me that I made time to watch them… outside of work…It was a very good use of time. … particularly just XX introduction to the field… and then … also XX talk on advance care planning I found particularly useful in addressing those common conversations about code status. (Resident)

Some residents were either unable to find the time or had to schedule time outside of work to engage in the curriculum. Even with our abbreviated curriculum, some participants were not able to engage in the extracurricular palliative education to the extent desired.

Last, *some attendings did not prioritize palliative care* (Subtheme 4):

There are certain attendings that… [are] like, whatever you wanna do… and then other attendings being a little more rigid. [They say] Well, that's not really something that we do. Let's just focus on, um, you know, the neurologic complaint (Resident)

I remember having a patient who… confide[d] in me about… her mood symptoms and started… crying during the visit because [she talked] about being estranged from her daughter… Well, this patient should probably be on an SSRI, and I remember asking about that. And one attending was like… we’re not gonna manage it, so we shouldn’t start it. And I was like… could we start it and refer? And they were like, no… [we]… didn’t even address that one thing that came up … it was… very unsatisfying. (Resident)

Residents must manage their patients with supervising attendings, so they were influenced by attendings who did not consider palliative care as part of their practice. One resident who advocated for palliative care wanted to address coping with illness but was stopped by her attending.

## Discussion

This study investigated the real-world implementation of a palliative care curriculum of lectures, note templates, and live meetings to neurology clinicians using surveys and interviews. Our findings indicate that the curriculum is desired, is feasible, and shows preliminary efficacy in teaching palliative care. However, our findings also highlight how barriers may make ideal implementation difficult and warrant further optimization.

This practice-based curriculum shows preliminary efficacy in teaching palliative care. Our surveys suggest that the multimodal curriculum may improve perceived knowledge of, comfort with discussing, and frequency of discussing palliative care topics. Some topics showed greater evidence of improvement (e.g., practical support for patients/care partners, symptom management, discussing disease course, and advance care planning) than others (e.g., spirituality and psychiatric concerns). One potential explanation is that the curriculum spent less time on spirituality and psychiatric concerns. It is also possible that these topics are inherently more challenging for residents because they delve into issues that may be uncomfortable to discuss or initiate. Consistent with this, the frequency of discussing these topics had smaller changes than learners' comfort and perceived knowledge. This could suggest that learners had fewer opportunities to practice these topics or that they needed additional instruction on how to initiate conversations around these topics.

However, barriers impeded the implementation of palliative care. Clinicians reported inadequate time when palliative issues arose, training to address the issues, or availability of timely clinical follow-up visits to discuss the issue further. Clinicians feared causing a bad scene and damaging clinical alliance with patients. These barriers of time, training, follow-up, and potential harm disincentivized clinicians from addressing palliative care. Serious illness communication can be viewed like other health care procedures,^18^ whose high therapeutic potential is accompanied by the potential to harm if performed poorly or even if in expert hands.

Furthermore, clinicians faced a conflict of hidden curriculum. When residents managed their clinical cases, some faculty were not interested in palliative discussions. They experienced a “hidden curriculum”^19^ phenomenon, where learners receive a formal curriculum teaching palliative care but encounter an informal, hidden curriculum in actual practice in resident clinic that is not supportive or even antagonistic toward palliative care. Attendings may reasonably feel that they can only teach what they know, having trained in a time when neurologists did not address palliative care. However, these disincentives to address palliative care are a loss for patients, residency training, and health care delivery at large.

Neurology clinicians want to address palliative issues but need education, practice, and clinical availability to do so. They need supervisors who are comfortable with discussing palliative care to align formal and informal curricula. Since this study, this curriculum has been integrated into resident education through academic half-days focused on neuropalliative care, featuring role-playing sessions with patient actors to practice giving bad news or addressing strong emotion. The curriculum is split into introductory lectures for newer learners and more advanced lectures for learners who were already exposed in previous years. At a larger, departmental level, we have recognized faculty who serve as champions of palliative care.

Study strengths include using quantitative and qualitative approaches and including multiple stakeholders including residents, APPs, and faculty.

Study limitations include a small sample size and incomplete data (nonpaired before education and after education surveys). The curriculum learners were different in the 2 cohorts. While the curriculum in 2021 was offered to learners who explicitly expressed interest in palliative care, the curriculum in 2022 was offered broadly to all first-time learners. This study did not measure the fidelity of curriculum components such as frequency of note template usage, live meeting attendance, or lectures watched. This study involved self-assessments rather than assessments of knowledge, behavior, or care.

This study's neurology department has a strong culture of neuropalliative care, which may limit generalizability of findings. Finally, there may be a response bias, as nonrespondents may be less interested in palliative care or have had a less positive experience with training.

This study shows that a palliative care curriculum can feasibly be implemented in real-world neurology settings and demonstrates preliminary efficacy. Neurology clinicians need ongoing education, supervisory support, and clinic structure to more optimally practice palliative care. Future studies are needed to better characterize mechanisms of curricula and implementation across diverse learners in different settings.

## Glossary

APP: advanced practice provider
